# Enhancement of solute diffusion in microdroplets using microrotors under rotational magnetic field

**DOI:** 10.1038/s41598-023-38299-z

**Published:** 2023-07-10

**Authors:** Shinji Bono, Kota Sakai, Satoshi Konishi

**Affiliations:** 1 Research Organization of Science and Technology, Ritsumeikan University, Kusatsu, 525-8577 Japan; 2 Ritsumeikan Advanced Research Academy, Kyoto, 604-8502 Japan; 3 Ritsumeikan Global Innovation Research Organization, Ritsumeikan University, Kusatsu, 525-8577 Japan; 4 Department of Mechanical Engineering, College of Science and Engineering, Ritsumeikan University, Kusatsu, 525-8577 Japan

**Keywords:** Fluidics, Fluids, Wetting

## Abstract

In vertical contact control (VCC), a microdroplet array selectively contacts with an opposite microdroplet array. Generally, VCC is useful for the dispenser mechanism based on solute diffusion between microdroplet pairs. However, sedimentation due to gravity can cause an inhomogeneous distribution of solutes in microdroplets. Therefore, it is necessary to enhance solute diffusion to achieve the accurate dispensing of a large quantity of solute in the direction opposite to that of gravity. Herein, we applied a rotational magnetic field to the microrotors in microdroplets to enhance the solute diffusion in microdroplets. Driven by microrotors, the rotational flow can generate a homogeneous distribution of solutes in microdroplets. We analyzed the diffusion dynamics of solutes using a phenomenological model, and the results showed that the rotation of microrotors can increase the diffusion constant of solutes.

## Introduction

Biochemical assays, such as polymerase chain reaction tests, have become increasingly important recently. To introduce the collected samples into the analytical instruments for these assays, samples are dispensed to well plates with test reagents. Dispensation is usually performed manually using a pipette. Therefore, this manual preparation, which poses a heavy burden on the operators and generates a large amount of waste plastic, must be replaced with an automatic and effective process.

Microdroplet arrays are desired for novel dispenser mechanisms^1,2^. For example, when we introduced a water solution to a wetting pattern substrate (where circular hydrophilic regions are patterned on a hydrophobic material), a microdroplet array can be formed spontaneously^3,4^. As microdroplets are separated from each other in an array, we can regard them as individual wells. In other words, a microdroplets array is a promising candidate for well plates that can be integrated onto a chip. In the hanging droplet method, biological cells are cultured in microdroplet arrays^2,5^.

After applying the vertical contact control (VCC) between a microdroplet array and an opposite array, microdroplets coalesce in pairs^6,7^. As solutes can travel through a coalescent microdroplet, the VCC of microdroplet arrays is an alternative manipulation approach for pipetting. By integrating electrowetting in the dielectric technique with VCC for manipulating the shape of microdroplets, it enables us to selectively control the contact between the arbitrary pairs of microdroplets^8,9^. A previous work reported that gravity can cause the transport of fluorescence beads (regarded as cells) to the bottom microdroplets through VCC^8^.

In addition, it is possible to transport solutes through coalescent microdroplets via diffusion. The test reagents initially introduced in the top microdroplets can diffuse through coalescent microdroplets during VCC and thus we can dispense test regents to the bottom microdroplets. A previous work observed cellular calcium oscillations after VCC between the bottom and top microdroplets, which include cells and histamine, respectively^10^. Moreover, the fluorescence reaction was reported to be controlled by the histamine concentration adjusted using the VCC of microdroplets.

The control of solute concentration in microdroplets is important for the biochemical application of the dispenser mechanism by the VCC of microdroplets. However, the density difference between the solute and water can cause a heterogeneous distribution of solute concentration in coalescent microdroplets along the direction of gravity. Although solute diffusion can partially reduce inhomogeneity, a homogeneous concentration distribution would not be achieved. The concentration difference between the solutes in the top and bottom microdroplets after separation depends on the solute diffusion properties. Therefore, an additional solute diffusion enhancement mechanism may enable us to obtain a homogeneous distribution of solute concentration, and it must contribute to the dispensation of solute from the bottom to the top microdroplets with an accurate concentration control.

The combination between a magnetic field and magnetized particles is attractive as a technique for the manipulation of targets in microdroplets. Previous work reported that the rotational magnetic field applied to paramagnetic particles in an aqueous solution on hydrophobic surfaces can enhance enzyme reaction in single droplets^11^. In addition, the application of magnetic manipulation to VCC of microdroplets was reported: Magnetic attractive force can transport magnetized particles against gravity^12^. Enhancement of material transportation between a pair of microdroplets using magnetic manipulation should enable us to homogenize the concentration of solutes and as a result, should achieve accurate concentration control.

Herein, we applied a rotational magnetic field to the microrotor incorporated in the microdroplets to enhance solute diffusion. The microrotors composed of magnetic materials demonstrate a unidirectional rotation under a rotational magnetic field. Consequently, they should drive the flow in microdroplets. We used flow as the enhancement mechanism of solute diffusion in microdroplets. Then, we investigated the effect of the rotational behavior of microrotors on solute diffusion and discussed the mechanism of diffusion enhancement.

## Results

**Figure 1:**
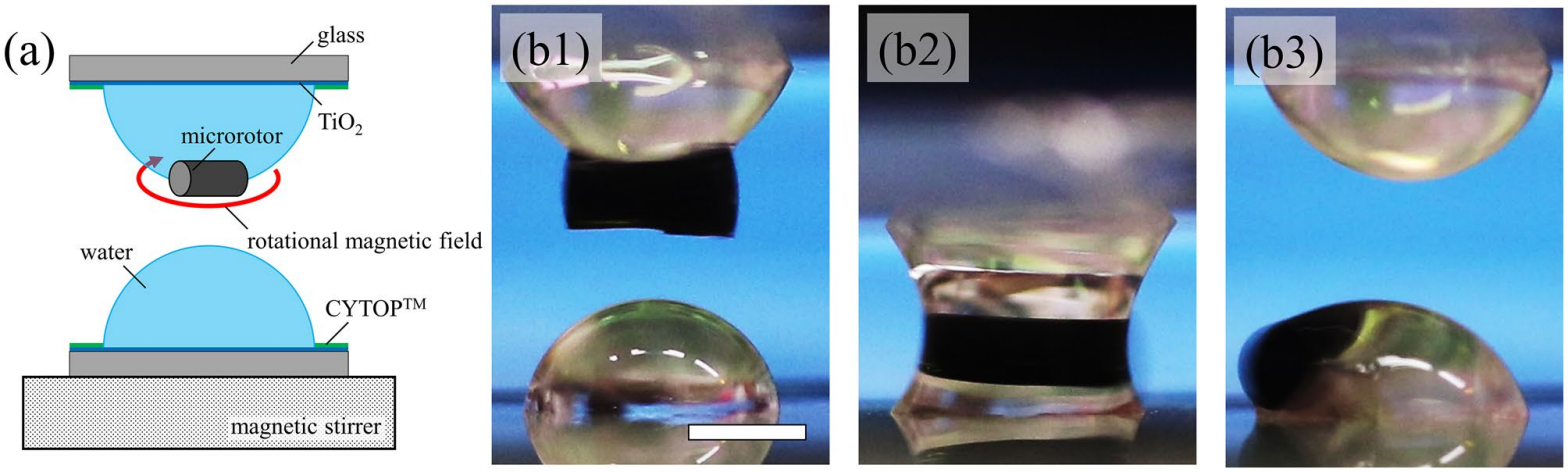
Side view of microdroplets with a microrotor through VCC. (**a**) Schematic of microdroplets before VCC. We introduced a microrotor in the top microdroplet. The magnetic stirrer below the bottom substrate generated a rotational magnetic field. (**b**) Side view of microdroplets through VCC: (**b1**) initial top microdroplet incorporating the microrotor under a rotational magnetic field from the magnetic stirrer; (**b2**) rotating microrotor in the coalescent microdroplet; and (**b3**) microrotor in the bottom microdroplet after separation. The scale bar indicates 1 mm.

We investigated the rotation behavior of microrotors in microdroplets under a rotational magnetic field. Figure 1a shows the schematics of the initial state of VCC. We used the wetting pattern substrates composed of hydrophilic and hydrophobic materials. Initially, we formed 4-µL water droplets with a radius of 1.24 mm on circular hydrophilic regions. We set a pair of wetting pattern substrates on the z-axis control stages to ensure that the top and bottom microdroplets are opposite to each other. A microrotor was incorporated in the top microdroplet. We set a magnetic stirrer below a bottom wetting pattern substrate and applied a rotational magnetic field to the microrotor in microdroplets.

We observed the VCC of microdroplets with a microrotor from the side, as shown in Fig. 1b1–b3 and Video 1 in Supplementary Information 1. Before the VCC, the microrotor rotated at the bottom of the top microdroplet, as shown in Fig. 1b1, because the density of the microrotor was larger than that of water. We lowered the top wetting pattern substrate with a rotational magnetic field and performed VCC for the pair of microdroplets, as shown in Fig. 1b2. We performed VCC of microdroplets while applying a rotational magnetic field to the microrotor in the top microdroplet. Rotational flow occurs in the coalescent microdroplet. On the other hand, the velocity of flow is zero at interfaces between substrates and water (stick condition). Since the velocity of flow at the center of the coalescent microdroplet is maximum, the microrotor rotated at the center of the coalescent microdroplet. After VCC for a period of *t* s with a rotational magnetic field, we separated the coalescent microdroplet into two hemispherical microdroplets. Wetting pattern substrates maintain the top microdroplet even after separation. Previous work reported that the morphology of coalescent microdroplets immediately before separation is determined by the force balance between gravity and surface tension^6^. Figure 1b3 shows the pair of microdroplets after separation. The microrotor was in the bottom microdroplet after VCC. Then, we removed the rotational magnetic field.

**Figure 2:**
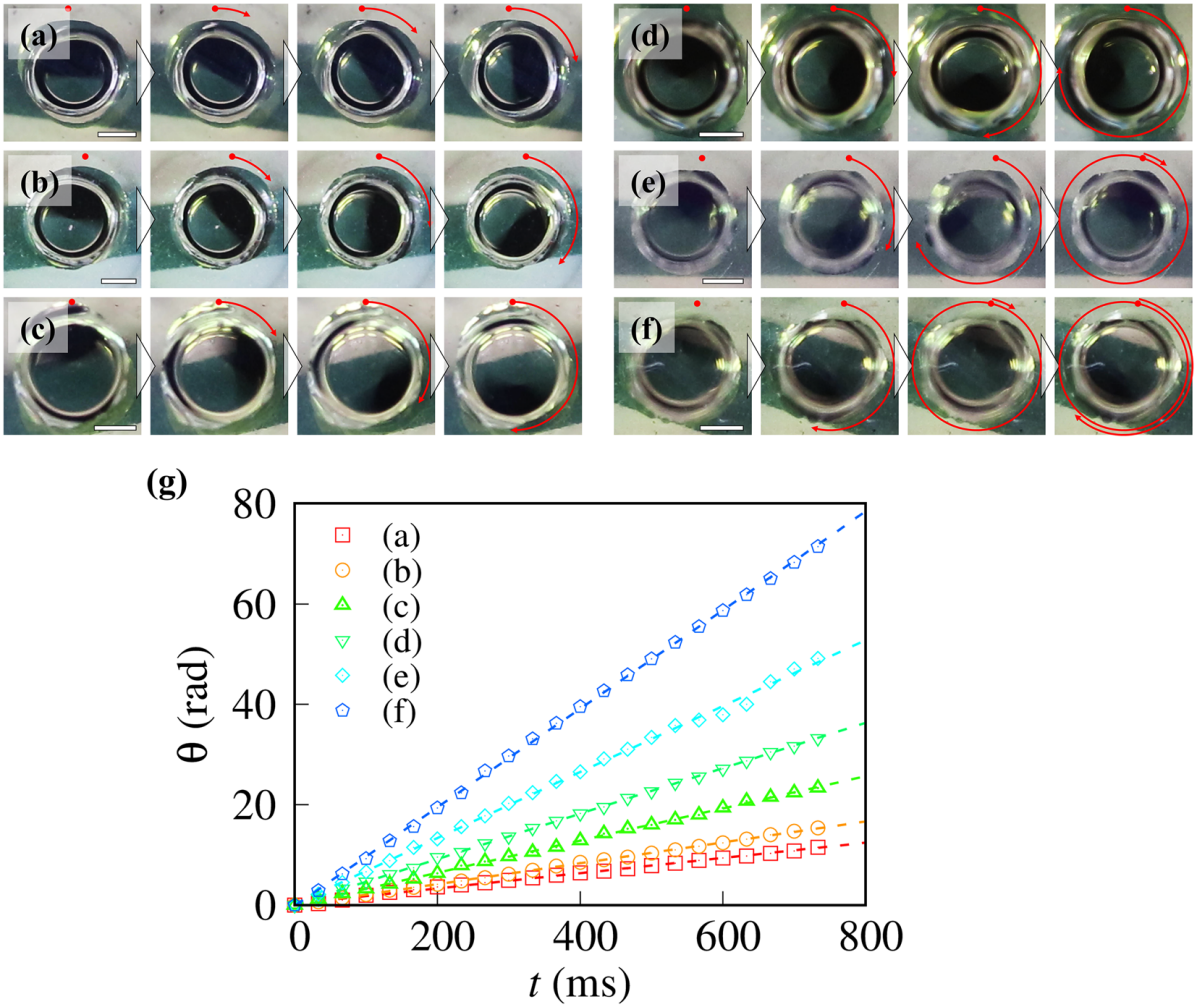
Rotation behavior of microrotors in the coalescent microdroplets. The top pictures show rotating microrotors with angular velocities of (**a**) 15 rad s^–1^, (**b**) 21 rad s^–1^, (**c**) 32 rad s^–1^, (**d**) 45 rad s^–1^, (**e**) 66 rad s^–1^ and (**f**) 98 rad s^–1^. These snapshots were taken every 33 ms. The scale bars indicate 1 mm. (**g**) The time evolution of the rotational angle θ of microrotors in the coalescent microdroplets. The dashed lines are the best-fit lines using the linear functions with respect to *t*.

Next, we modulated the rotation speed of the microrotors in the coalescent microdroplets by adjusting the frequency of the rotational magnetic field. To quantitatively evaluate the rotation speed, we observed the rotating microrotors in the coalescent microdroplets from the top. Figure 2a–f and Video 2a–f in Supplementary Information 1 show the top view of the rotation behavior of the microrotors under an external magnetic field modulated in six steps. Microrotors always rotate in a clockwise direction, which agrees with the direction of the external rotational magnetic field. We measured the rotation angle θ of microrotors, as shown in red arrows in Fig. 2a–f. Figure 2g shows the time evolution of θ. From our observations, θ depends on *t* linearly. We obtained the angular velocity ω by fitting the experimental data using linear functions. Accordingly, we found that ωs of the microrotors in Fig. 2a–f was found to be 15, 21, 32, 45, 66, and 98 rad s^–1^, respectively.

**Figure 3:**
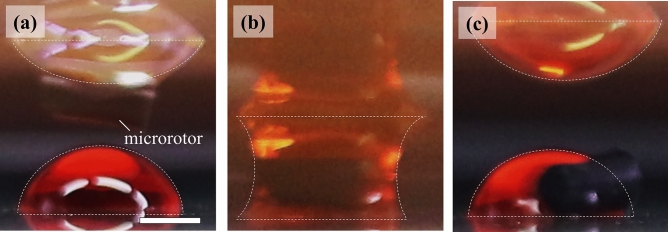
Effect of the rotation of microrotors on the diffusion of dye in the coalescent microdroplet (Video 2 Supplementary Information). (**a**) Side view of the initial microdroplets. We incorporated a microrotor and red dye into the top and bottom microdroplets, respectively. The microrotor continuously rotated with an angular velocity of 15 rad s^–1^ under a rotational magnetic field. (**b**) Side view of the coalescent microdroplet. We maintained contact between the microdroplets for *t* s. (**c**) Side view of the separated microdroplets after VCC. The scale bar indicates 1 mm. The interfaces between the microdroplets and air/substrates are highlighted with white dashed lines.

We then investigated the effect of rotating microrotors on the diffusion phenomena in the microdroplets. As shown in Fig. 3a, we initially incorporated the microrotor and red dye into the top and bottom microdroplets, respectively. Therefore, the colors of the top and bottom microdroplets were transparent and red, respectively. Then, we applied a rotational magnetic field to enable the continuous rotation of the microrotor. Herein, we focused on the diffusion of the red dye from the bottom to the top of the microdroplets, that is, in the opposite direction of gravity.

After the initial formation of the microdroplets, we lowered the top microdroplet and performed the VCC for a pair of microdroplets, as shown in Fig. 3b. The pair of microdroplets contacted each other for a period of *t* s with the rotation of the microrotor. The color of the coalescent microdroplet became homogeneously distributed over time, indicating that the red dye can diffuse in the coalescent microdroplet through VCC. Figure 3c shows the separated microdroplets after the VCC. The red color of the top microdroplet suggested that the red dye was transported in the opposite direction to gravity due to diffusion.

**Figure 4:**
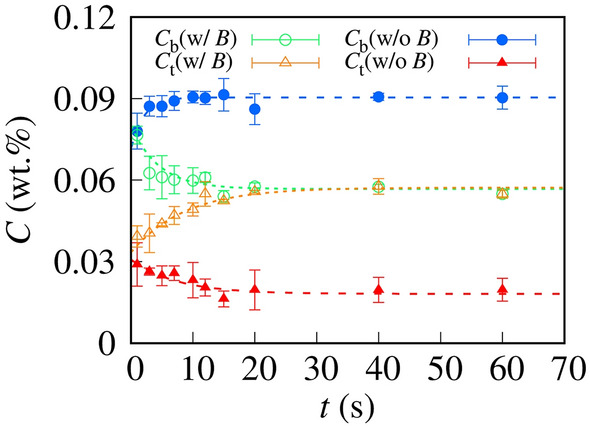
Dye concentration in microdroplets as a function of the contact time *t* after VCC. *C*_t_ and *C*_b_ are the concentrations in the top and bottom microdroplets, respectively; w/*B* and w/o *B* denote the concentrations with and without the rotational magnetic field, respectively. The angular velocity of the microrotor was 15 rad s^–1^. The dashed lines are the best fits obtained using single-exponential functions.

After the VCC, we measured the concentration of the red dye in the microdroplets. We collected the separated microdroplets, as shown in Fig. 3c, by pipetting and then placed them in a microvolume UV–Vis spectrophotometer. We measured the absorbance at 350 nm and obtained the concentration of the red dye according to the Beer–Lambert law (Supplementary Information 1)^13,14^. Figure 4 shows the concentration of the red dye as a function of the contact time *t*. We define the concentration of the red dye in the top and bottom microdroplets as *C*_t_ and *C*_b_, respectively. w/*B* and w/o *B* indicate the concentrations with and without the rotational magnetic field, respectively.

Without the magnetic field (w/o *B*), *C*_b_ and *C*_t_ in the steady state converged to 9 × 10^–2^ and 2 × 10^–2^ wt.%, respectively. We define the concentration difference as ∆*C* = *C*_b_ − *C*_t_ and ∆*C* (w/o *B*) is 7.2 × 10^–2^ wt.%. The positive ∆*C* corresponds to the inhomogeneous distribution caused by the sedimentation of the dye, resulting from the density difference between the dye and water.

Shortly after the VCC, the microrotor as well moved from the top microdroplet to the bottom one. Transient flow occurred shortly after the VCC enhanced diffusion, and therefore, ∆*C* at *t* < 10 s was smaller than that in the steady state stage. As the transient flow would disappear in the steady state, ∆*C* converges on the constant value determined by the density difference.

Meanwhile, without the rotational magnetic field (w/o *B*), *C*_b_ and *C*_t_ monotonously decreased and increased, respectively. After 20 s, *C*_b_ became equal to *C*_t_ (~ 5 × 10^–2 ^wt.%). This result showed that enhanced dye diffusion through a microrotor under a rotational magnetic field achieved a homogeneous concentration distribution. In other words, the incorporation of microrotors enabled us to transport solutes homogeneously in the direction opposite to that of gravity.

## Discussion

**Figure 5:**
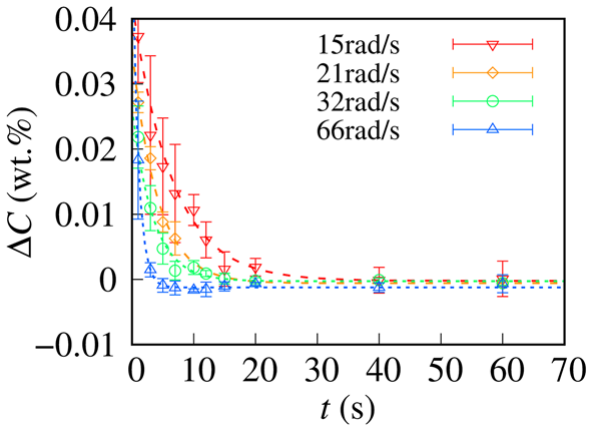
Time evolution of ∆*C* = *C*_b_ − *C*_t_. The dashed lines are the best-fit curves obtained using the single-exponential function, Eq. (1). The obtained τs are 6.4 ± 0.7, 4.1 ± 0.5, 3.4 ± 0.4, and 1.0 ± 0.3 s for angular velocities of 15, 21, 32, and 66 rad s^–1^, respectively.

To reveal the effect of the rotation of microrotors on dye diffusion, we investigated the dynamics of dye diffusion in the coalescent microdroplets. Figure 5 shows the time evolution of ∆*C*. Under the rotational magnetic field, ∆*C* converged to ~ 0 wt.% under our experimental conditions, suggesting that the use of microrotors enabled us to achieve a homogeneous distribution for the dye concentration in microdroplets.

The relaxation time of ∆*C* decreased with increasing ω. We fitted the experimental data with single-exponential functions to obtain the relaxation time τ.

1 $$\Delta C = C_{0} {\text{exp}}\left( { - t/\uptau } \right),$$

where *C*_0_ is the initial concentration. The single-exponential functions were found to agree with the experimental data.

**Figure 6:**
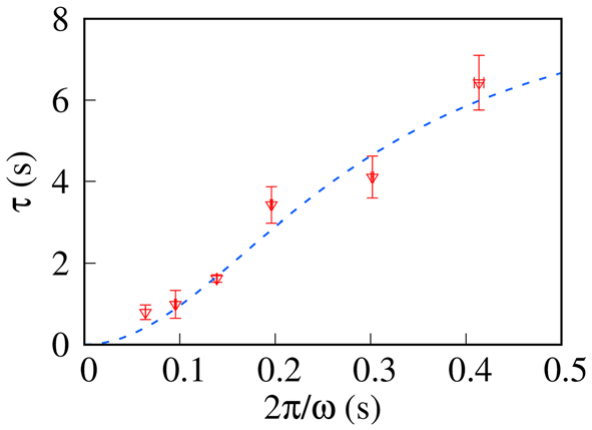
Relaxation time τ of microrotors as a function of the rotational period (2π/ω). The dashed line is the best-fit curve obtained using the Lorentz function, Eq. (6). From the fitting results, *d*_0_ and *d*_2_ are (1.1 ± 0.3) × 10^−1^ and (9.3 ± 1.1) × 10^−3^ s, respectively.

We summarize the fitted τ in Fig. 6. Here, we define the rotation period of microrotors as 2π/ω. We found that τ monotonously decreased with decreasing 2π/ω.

The effect of rotating microrotors on diffusion dynamics was discussed using a phenomenological model. The application of an external magnetic field drives the rotation of microrotors, which causes flow in the coalescent microdroplet. Viscous dissipation converts the kinetic energy of the rotating microrotor to both heat and mixing entropy of solutes, which enhances the diffusion of solutes. To construct a phenomenological model, we normalize the effect of the enhancement of the microrotor into diffusion constant.

The diffusion equation of the red dye in the coalescent microdroplets is given as

2 $$\frac{\partial C}{\partial t}\text{ = }{D}\frac{{\partial }^{2}C}{\partial {x}^{2}},$$

where *D* is the diffusion constant. We assumed that the distribution of the dye concentration changes with the characteristic wave number *k* (~ 1/λ, where λ is a characteristic length scale). Then, the diffusion equation of ∆*C* is approximately given as^15,16^

3 $$\frac{\partial }{\partial \it{t}}\Delta {C}\text{ }- \, {\it{D}}\frac{\Delta {\it{C}}}{{\uplambda }^{2}}.$$

Substituting Eq. (1) into (3), we obtain the relaxation time as follows:

4 $$\frac{1}{\uptau }\text{ = }\frac{\it{D}}{{\uplambda }^{2}}.$$

To include the effect of the rotation of the microrotors in our model, we considered the ω dependence of the diffusion constant *D*. Mirror symmetry requires that *D* is independent of the inversion of the rotational direction (ω → – ω). Thus, we phenomenologically represent ω dependence of *D* as

5 $$D(\upomega ) = D_{0} + D_{2} \upomega^{2} + O(\upomega^{4} ),$$

where *D*_0_ and *D*_2_ are the coefficients of the zero-order and second-order of ω, respectively. Substituting *D*(ω) in Eq. (5) into (4), we obtain the ω dependence of τ as follows.

6 $$\uptau = \frac{1}{{d_{0} + d_{2} \upomega^{2} }}.$$

Here, we use the normalized parameters *d*_0_ = *D*_0_/λ^2^ and *d*_2_ = *D*_2_/λ^2^. This suggests that τ is the Lorentz function of ω.

To compare the theoretical and experimental results, we fitted the experimental results using Eq. (6), as shown in the dashed line in Fig. 6. Our theoretical prediction agreed with the experimental results, which suggested that the rotation of microrotors enhanced solute diffusion.

**Figure 7:**
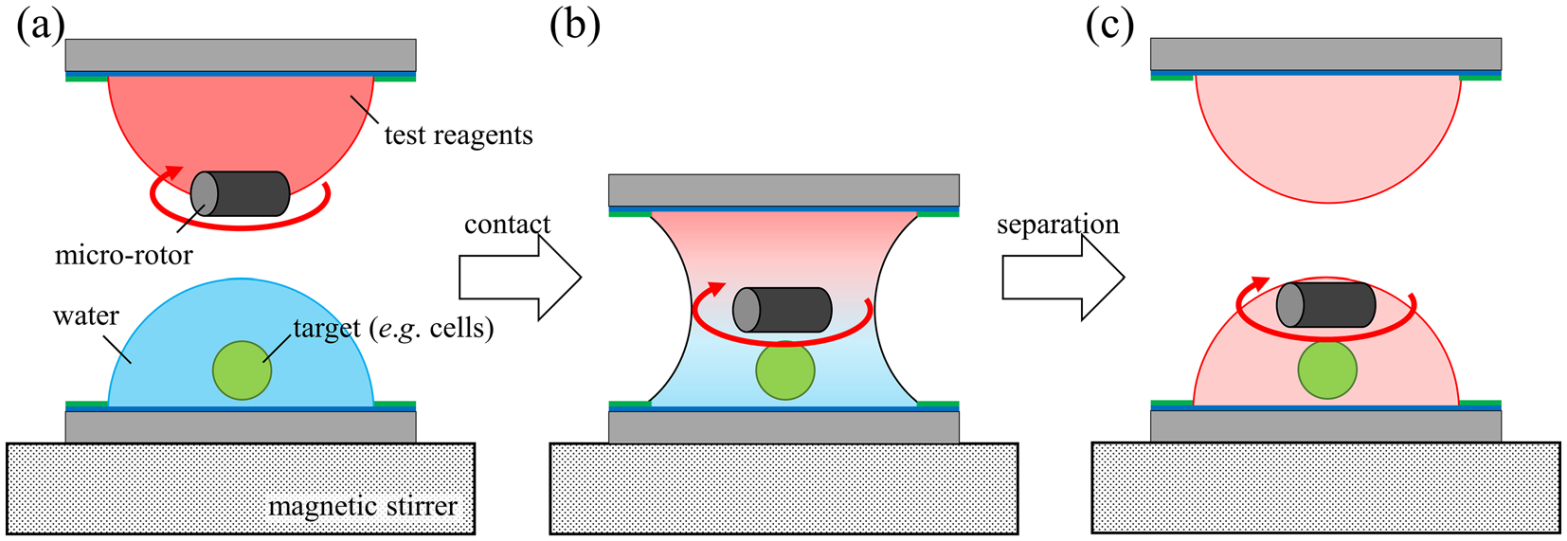
Schematics of application of microrotors under rotational magnetic field to biochemical assay. (**a**) Initial state of VCC of microdroplets. Test reagents and a microrotor were introduced in top microdroplet. On the other hand, tagets such as cells were introduced in bottom microdroplet. (**b**) Enhancement of solute diffusion in a coalescent microdroplet using microrotor. (**c**) Microdroplets after separation. Microrotor under rotational magnetic field achieves uniform concentration of test reagent.

Previously we succeeded in injecting chemical reagents into cells using VCC of microdroplets. To apply this technique to biochemical assay, transportation of solutes against gravity is essential. Our findings achieve uniform distribution of solute concentration and acceleration of diffusion. Figure 7 shows the concept of the application using microrotors under a rotational magnetic field. Initially, test reagents and cells are introduced into top and bottom microdroplets as well as microrotor. Test reagents diffuse while VCC. Then, the coalescent microdroplet is separated to complete the injection of test reagents. The inhomogeneity of test reagents due to gravity prevents us from predicting the concentration quantitatively in advance. On the other hand, the usage of microrotors realizes uniform distribution of solute concentration and as a result, achieves accurate concentration control, where the concentration must equal half of the initial concentration. Therefore, our findings must contribute to accurate concentration control in biochemical assays.

In this study, we succeeded in enhancing the diffusion of solutes in microdroplets using microrotors under a rotational magnetic field. Here, we investigated the diffusion dynamics of solutes while focusing on rotational velocity. Besides, the size of the microrotor must affect the diffusion of solutes. For example, the miniaturization of microrotors should decrease the effect of enhancement of solute diffusion. The effect of the size of the microrotor should be investigated in the future.

## Materials and methods

### Initial microdroplets on the wetting pattern substrates

We patterned hydrophilic TiO_2_ and hydrophobic CYTOP™ on glass substrates. We fabricated wetting pattern substrates following the process reported in a previous study^6^. We pipetted pure water on the top wetting pattern substrates and formed microdroplets initially. An aqueous solution with a red dye (New Coccine, FUJIFILM Wako Pure Chemical Co.) of 1.0 × 10^–1^ wt.% was pipetted to form microdroplets on the wetting pattern substrates. We designed the initial volume of microdroplets *V* and the radius of circular hydrophilic regions *R* to be 4 μL and 1.24 mm, respectively. As *V* = 2π/3 *R*^3^, hemispherical microdroplets were initially formed on the wetting pattern substrates with a contact angle of 90°.

### Fabrication process of microrotors

**Figure 8:**
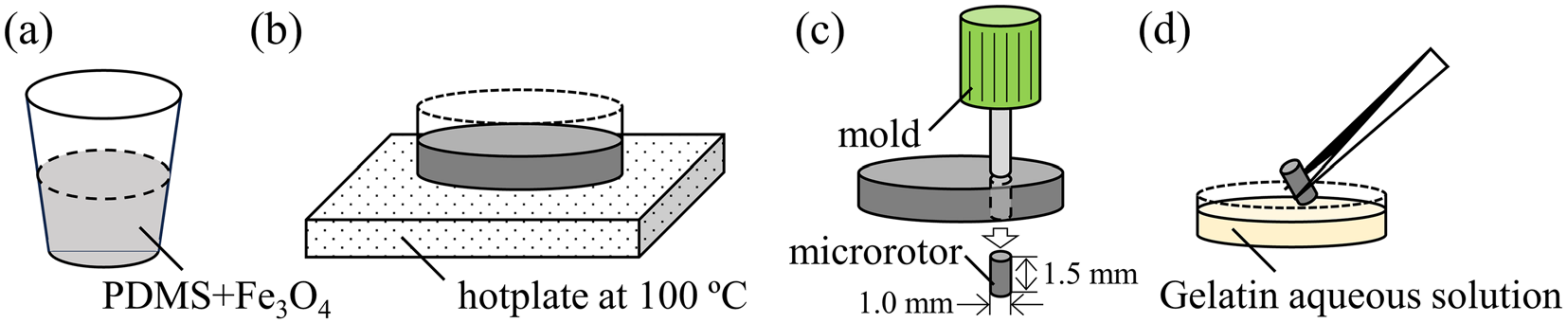
Fabrication process of microrotors. (**a**) Mixture of PDMS and Fe_3_O_4_. (**b**) Heat-cure process of the mixture at 100 °C. (**c**) Shaping process of microrotor using a mold. (**d**) Surface treatment of microrotor with Gelatin aqueous solution.

To obtain the microrotors, we mixed 71.4 wt.% of polydimethylsiloxane (PDMS, DuPont Toray Specialty Materials K.K.) and 28.6 wt.% of Fe_3_O_4_ (NAKARAI TESQUE, INC.) (Fig. 8a). We heat-cured the mixture (with a thickness of 1.5 mm) at 100 °C for 60 min (Fig. 8b). We shaped the cylindrical microrotors (diameter ϕ = 1.0 mm and length *d* = 1.5 mm) out of the heat-cured mixture with the mold (Fig. 8c), where we used cutaneous biopsy punch (KAI INDUSTRIES CO., LTD.). To obtain the hydrophilic surface of PDMS, we dipped the cylindrical microrotors in 5 wt.% of a Gelatin (NAKARAI TESQUE, INC.) aqueous solution and then dried the microrotors (Fig. 8d). This paper focused on proving the concept of diffusion enhancement, and thus, optimizing the design of microrotors was not considered. The microrotors were subjected to a rotational magnetic field generated by a magnetic stirrer (SRS116AA, KENIS Ltd.).

### Spectroscopy for determining dye concentration in microdroplets

A microvolume UV–Vis spectrophotometer (NanoDrop One, Thermo Fisher Scientific Inc.) was used to estimate the dye concentration in microdroplets. In our experiment, the concentration in microdroplets should be less than the initial dye concentration (~ 1.0 × 10^–1^ wt.%). Then, we performed calibration and confirmed that the absorbance at 350 nm was proportional to the concentration under our experimental condition (Supplementary Information 1).

## Conclusion

Herein, we enhanced the dye diffusion in microdroplets using microrotors under a rotational magnetic field and achieved homogeneous mixing within a few seconds. The stable and continuous rotation of microrotors in microdroplets was driven by the rotational magnetic field. We investigated the diffusion phenomena of solutes from the bottom to the top microdroplets through VCC. As the density of the solutes was larger than that of water, the concentration of the solutes in the bottom microdroplets was higher than that in the top microdroplets after VCC. Meanwhile, the microrotors under a rotational magnetic field enabled us to achieve a homogeneous distribution of the solute concentration. Therefore, the enhancement of solute diffusion in the direction opposite to that of gravity is achievable with our mixing mechanism.

The concentration difference between the top and bottom microdroplets decreased exponentially with the contact time. The relaxation time of homogeneous mixing accelerated monotonously with the increasing angular velocity of microrotors. For quantitative estimation, we analyzed the experimental results using a phenomenological model, where the angular velocity in the diffusion constant is normalized. This simple model predicts that the relaxation time is given by the Lorentz function of the angular velocity, which agrees with the experimental results. In general, the rotation of microrotors can increase the diffusion constant of the solutes to enhance solute diffusion. Our novel mechanism of diffusion enhancement can contribute toward achieving an accurate concentration control in biochemical assays using microdroplets.

## Supplementary Information

Supplementary Video 1.

Supplementary Video 2.

Supplementary Video 2.

Supplementary Video 2.

Supplementary Video 2.

Supplementary Video 2.

Supplementary Video 2.

Supplementary Video 3.

Supplementary Information.

## Data Availability

The data that support the findings of this study are available from the corresponding author upon reasonable request.
