# Supplementary material for: Enhancement of solute diffusion in microdroplets using microrotors under rotational magnetic field
Source: Sci Rep. 2023 Jul 10;13:11169. doi: 10.1038/s41598-023-38299-z (PMC10333317; doi:10.1038/s41598-023-38299-z)
Supplement: Supplementary file 9 — Supplementary Information. [file 41598_2023_38299_MOESM9_ESM.docx]

Supplementary Information for

**Enhancement of solute diffusion in microdroplets using microrotors under rotational magnetic field**

Shinji Bono*, Kota Sakai, and Satoshi Konishi

*Corresponding author. Email: [bono@fc.ritsumei.ac.jp](mailto:bono@fc.ritsumei.ac.jp)

**Supplementary Information 1: Calibration of the relation between concentration and absorbance**

The initial concentration of the red dye is 0.1 wt.%. For calibration, we measured the absorbance in a red dye aqueous solution with concentrations of 0, 0.025, 0.05, and 0.1 wt.%. Figure S1 shows the *C*_dye_ dependence of absorbance, where *C*_dye_ is the bulk concentration of red dye. Absorbance was shown to be proportional to *C*_dye_, which agrees with the Beer–Lambert law. For a quantitative evaluation, we fitted the experimental results using the following linear function:

(Absorbance) = α_S_ *C*_dye_ + β_S_. (S1)

The fitting parameters α_S_ and β_S_ were obtained to be 1.04 × 10^2^ wt.%^−1^ and 2.9 × 10^−2^, respectively. We can estimate the concentration of the red dye in the microdroplets using Eq. (S1).


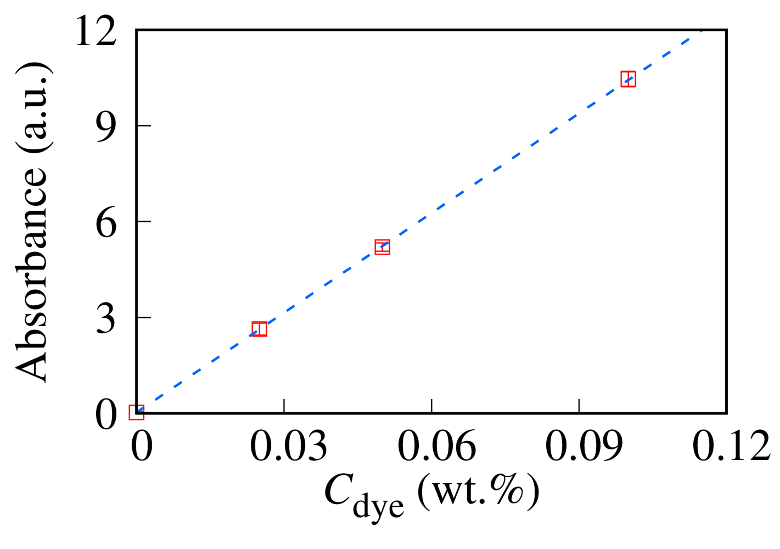


**Figure S1.** Relation between *C*_dye_ and absorbance. The dashed line is the best-fit line obtained using the linear function, Eq.(S1).

**Supplementary Information 2: Description of the supplementary video**

**Supplementary Video 1:**

The video corresponds to Fig.1. We performed VCC by applying a rotational magnetic field to the microrotor in a coalescent microdroplet. The scale bar indicates 1 mm.

**Supplementary Video 2 (a–f):**

Video 2 (a–f) corresponds to the results in Fig. 2 (a–f), respectively. The top view video of the rotating microrotors in the coalescent microdroplets. The recording was performed at three times slower speed.

**Supplementary Video 3:**

The videos correspond to the results in Fig. 3. The scale bar indicates 1 mm.
